# A stent of strength: use of lumen-apposing metal stents (LAMS) for biliary pathologies and other novel applications

**DOI:** 10.1007/s00261-024-04561-9

**Published:** 2024-09-10

**Authors:** Inessa Goldman, Katherine Ji, Meir H. Scheinfeld, Kaveh Hajifathalian, Matthew Morgan, Julie Yang

**Affiliations:** 1 Rutgers, The State University of New Jersey, Newark, USA; 2 Montefiore Medical Center, The Bronx, USA; 3 University of Pennsylvania, Philadelphia, USA; 4 Present Address: Rutgers, The State University of New Jersey, New Brunswick, USA

## Introduction

Lumen-apposing metal stents (LAMS) have emerged as the preferred method for minimally invasive drainage of symptomatic peripancreatic collections, including the management of infected walled-off necrosis (WON) [1]. The adoption of LAMS for the treatment of peripancreatic collections is due to several unique design features including broad anchoring flanges and shorter length, which allow for close apposition between a collection wall and gastrointestinal tract lumen, thereby reducing stent migration, leakage, and associated adverse events. The wide luminal diameter of LAMS, compared to plastic or metal biliary stents facilitates drainage of solid debris and serves as access for direct endoscopic necrosectomy. LAMS consists of nitinol with full silicone covering, which facilitates stent removal and mitigates tissue ingrowth. Finally, the development of an electrocautery-enhanced delivery system (Hot Axios™, Boston Scientific, Marlborough, MA) allows a one-step stent deployment and drainage [2].

Although the primary FDA-approved indication for LAMS use is drainage of symptomatic peripancreatic fluid collections, in 2023, they gained FDA approval for endoscopic gallbladder drainage in acute cholecystitis for high risk surgical candidates. Due to their versatility, LAMS are increasingly utilized by advanced endoscopists in various other novel off-label abdominopelvic applications, including biliary drainage in malignant distal biliary obstruction, gastric access temporary for endoscopy (GATE) in altered or post-surgical anatomies including Whipple or Roux-en-Y reconstructions, management of luminal GI strictures, enteric bypass with the creation of gastroenterostomies, and drainage of abdominal and pelvic collections, among others.

The successful expansion of LAMS applications and management of associated adverse events depend on the local expertise and creativity. There is a paucity of radiologic literature regarding the imaging appearance and anticipated utilizations and complications of LAMS. As novel applications for LAMS continue to grow, radiologists’ awareness is essential to detail the anatomical structures for optimal LAMS placement, plan and confirm acceptable placement, and rapidly identify possible adverse events. In this manuscript, we provide imaging examples of creative utilization and potential adverse events of LAMS in clinical practice.

## Endoscopic gallbladder drainage

The definitive therapy for acute cholecystitis (AC) is laparoscopic cholecystectomy (CCY). However, in cases of AC in unstable patients, immediate cholecystectomy may be challenging or contraindicated due to the extent of inflammation, high surgical risk factors, or organ dysfunction [3–5]. Current guidelines recommend percutaneous cholecystostomy (PC) for high-risk surgical patients and as the preferred modality in patients unable to tolerate general anesthesia [3–6]. However, indefinite maintenance of a PC can be uncomfortable, and is linked to adverse events (AEs) such as catheter dislodgement and infection, requiring frequent reintervention in 25–66% of patients [4, 7, 8].

**Fig. 1:**
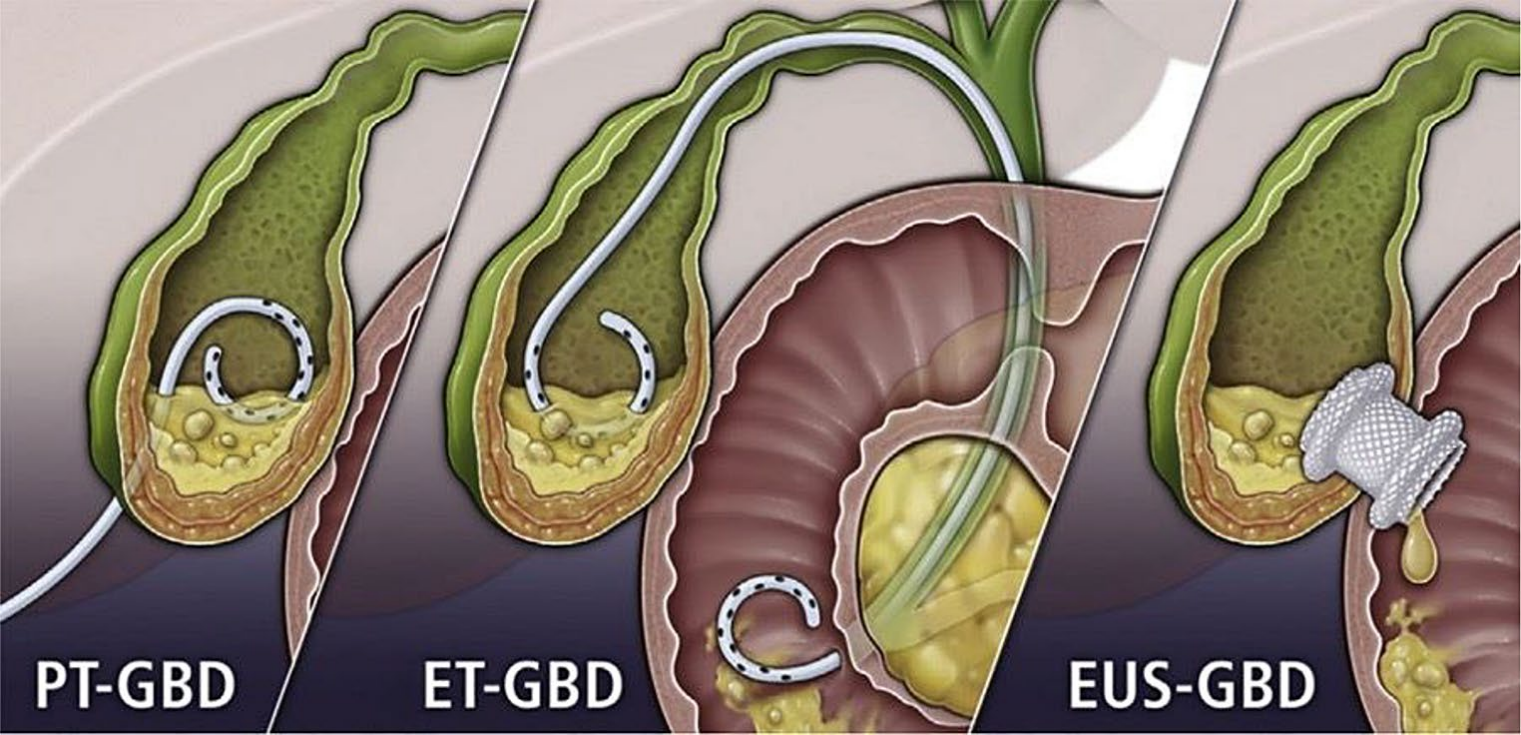
Gallbladder drainage procedures including percutaneous cholecystostomy (PC), endoscopic transpapillary gallbladder drainage (ET-GBD), and EUS-guided GBD (EUS-GBD). (Reprint from Gastrointest Endosc 2021;93:797–804, with permission

Endoscopic gallbladder drainage procedures, including endoscopic transpapillary gallbladder drainage (ET-GBD) and endoscopic ultrasound-guided gallbladder drainage (EUS-GBD) [9] have emerged as alternative treatment options (Fig. 1). ET-GBD involves placement of a stent from the duodenum into the gallbladder via the major papilla and common bile duct, making it suitable for patients with choledocholithiasis or cholangitis who may require simultaneous ERCP. However, ET-GBD faces many endoscopic technical challenges in identifying and cannulating a tortuous cystic duct, particularly in cases of large stone burden [10].

**Fig. 2:**
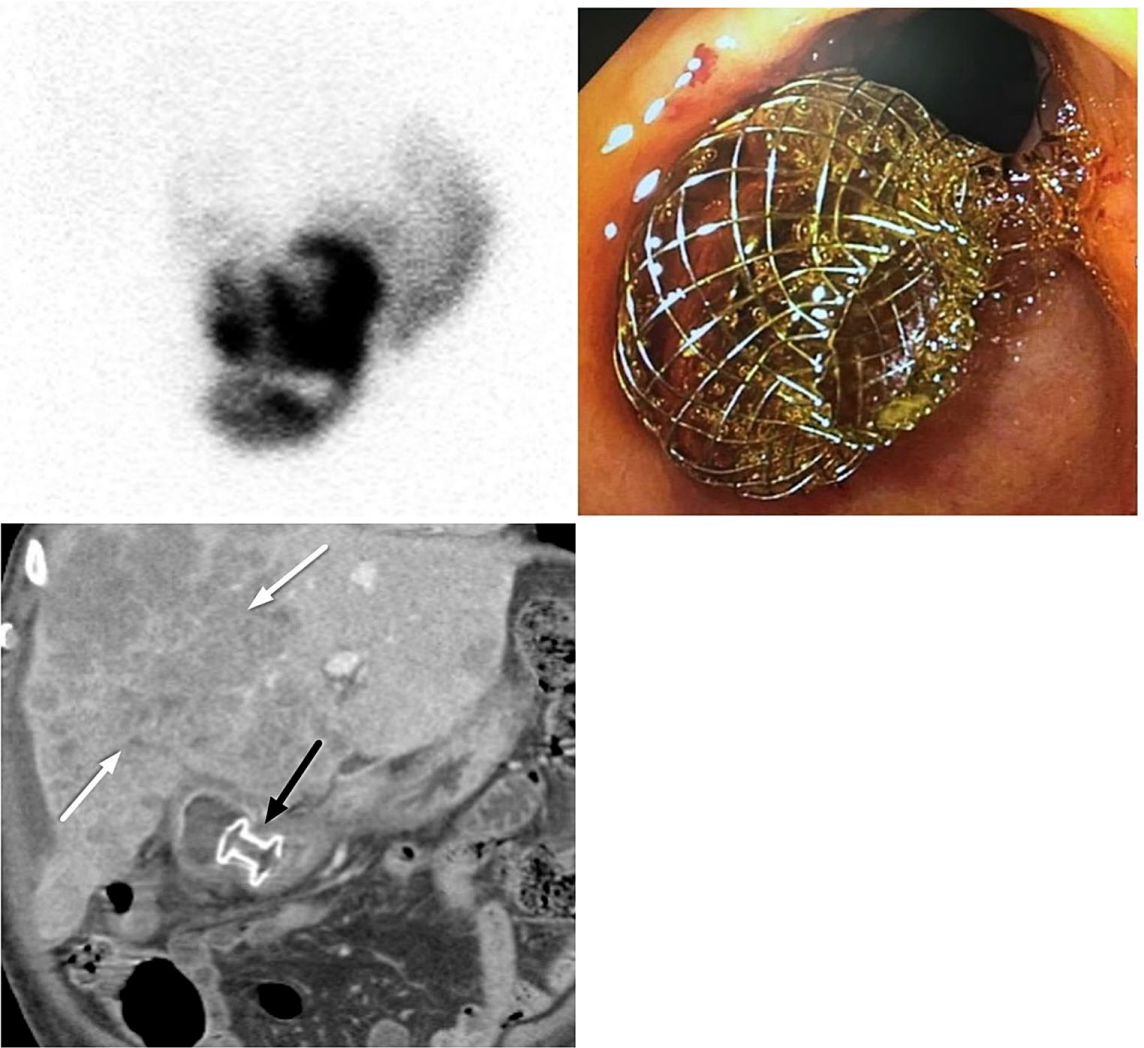
79 year-old male with prostate cancer, a poor surgical candidate, presented with acute cholecystitis and Klebsiellae bacteremia. **A**. HIDA scan at 4 h shows no tracer excretion into the gallbladder, diagnostic of acute cholecystitis. **B**. Endoscopic image from gastric antrum demonstrates LAMS cholecystogastrostomy was performed as an alternative to surgery and percutaneous cholecystostomy. **C**. Contrast-enhanced coronal CT image demonstrates extensive hepatic metastases (white arrows) precluding percutaneous drainage. Endoscopic cholecystogastrostomy utilizing LAMS (black arrow) was successfully performed as a therapeutic alternative

In EUS-GBD, the gallbladder is drained directly into the stomach (cholecystogastrostomy) or duodenum (cholecystoduodenostomy) under EUS guidance. LAMS have become the stent of choice, owing to their simplified electrocautery-enhanced deployment and dumbbell-shaped design, which improves gallbladder and bowel wall apposition, reducing leakage, perforation, and stent migration. The short and wide saddle (10–15 mm midsection) of LAMS reduces stent obstruction from stones, sludge, and debris, and allows for various additional therapeutic maneuvers through LAMS including cholecystoscopy, stone extraction, lithotripsy, and polypectomy [11–13]. Because of these unique characteristics, LAMS may be utilized for endoscopic gallbladder drainage in patients who are high-risk for surgery, in cases of failed percutaneous drainage, or in palliative situations. For example, we demonstrate one successful case of EUS-GBD using LAMS in a patient with acute cholecystitis in whom percutaneous drainage was precluded due to extensive hepatic metastases (Fig. 2).

**Fig. 3:**
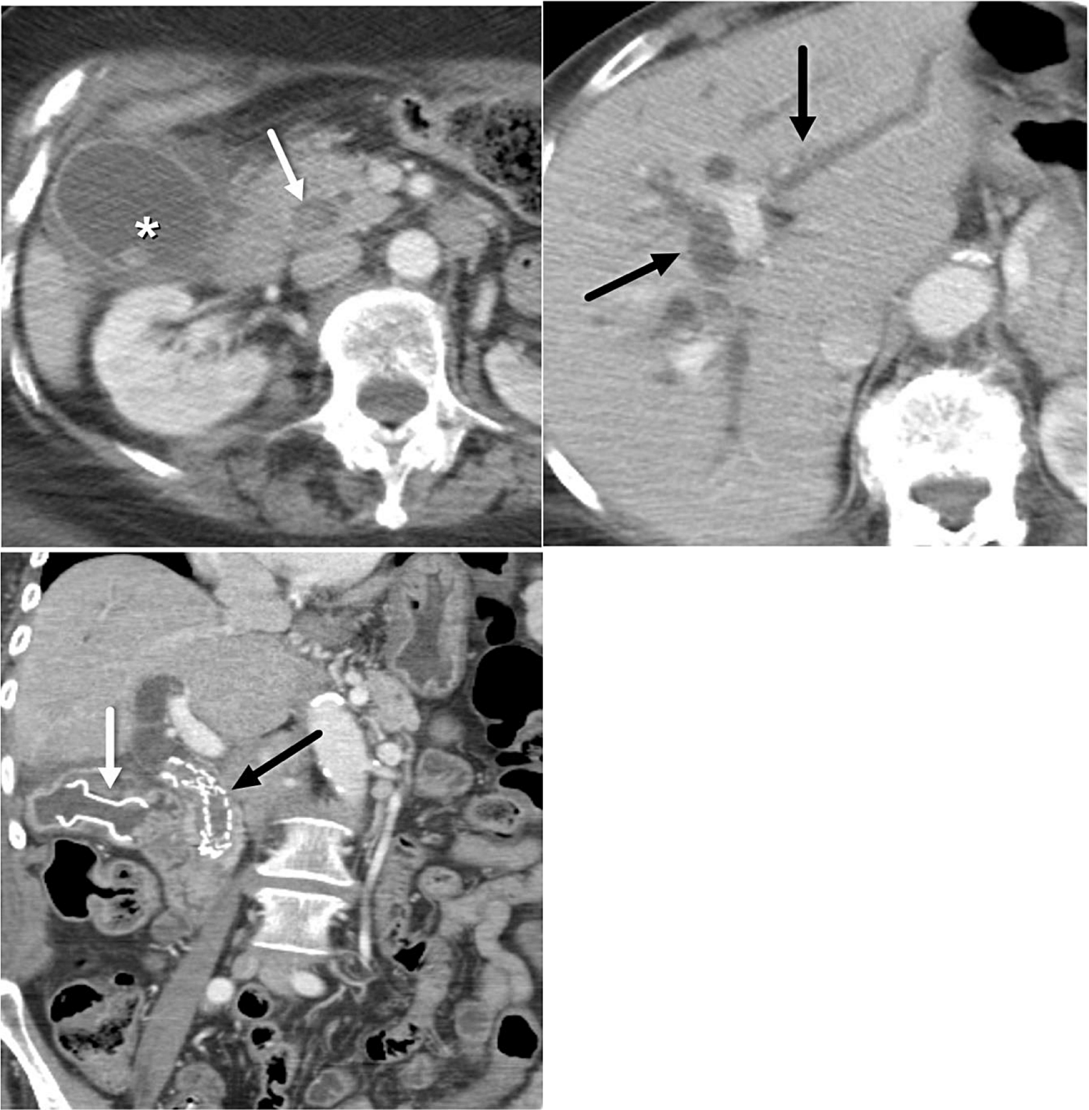
86 year-old female presented with acute cholecystitis complicated by ascending cholangitis due to choledocholithiasis. **A.** Contrast-enhanced axial CT image shows cholelithiasis, gallbladder wall thickening, and pericholecystic fluid of acute cholecystitis (white asterisk) complicated by ascending cholangitis due to choledocholithiasis (white arrow). **B.** Contrast-enhanced axial CT demonstrates moderate bilobar intrahepatic biliary dilatation (black arrows). **C.** Contrast-enhanced coronal CT image shows LAMS (white arrow) draining the gallbladder into the duodenum and a biliary stent (black arrow) placed during the same ERCP session in this patient who was a poor surgical candidate. Both the biliary stent and LAMS remained in place for 5 years. The patient had one subsequent bout of cholangitis and ultimately passed away at the age of 92 of unrelated causes

The optimal indwelling time for LAMS within the gallbladder has not been established. Removal of LAMS from the gallbladder generally leads to spontaneous closure of the cholecystogastrostomy and recurrent cholecystitis unless the cystic duct obstruction is resolved. On the other hand, if a stent remains indefinitely, stent migration or gallbladder wall erosive injury and bleeding of the gallbladder/stomach/duodenal wall may occur. Long-term stent placement, up to 3 years, has been reported without stent-related adverse events [14]. There are anecdotal cases of longer adverse event-free implantation times, such as we have demonstrated (Fig. 3).

While multiple retrospective cohort studies and meta-analyses have demonstrated comparable technical and clinical success rates for both endoscopic gallbladder drainage techniques (ET-GBD, and LAMS EUS-GBD) and PC, the endoscopic gallbladder drainage techniques have demonstrated lower rates of adverse events and improved quality of life [15–20]. Teoh et al. demonstrated significantly reduced 30-day adverse events (12.8% vs. 47.5%), and recurrent cholecystitis (2.6% vs. 20%) [21].

**Fig. 4:**
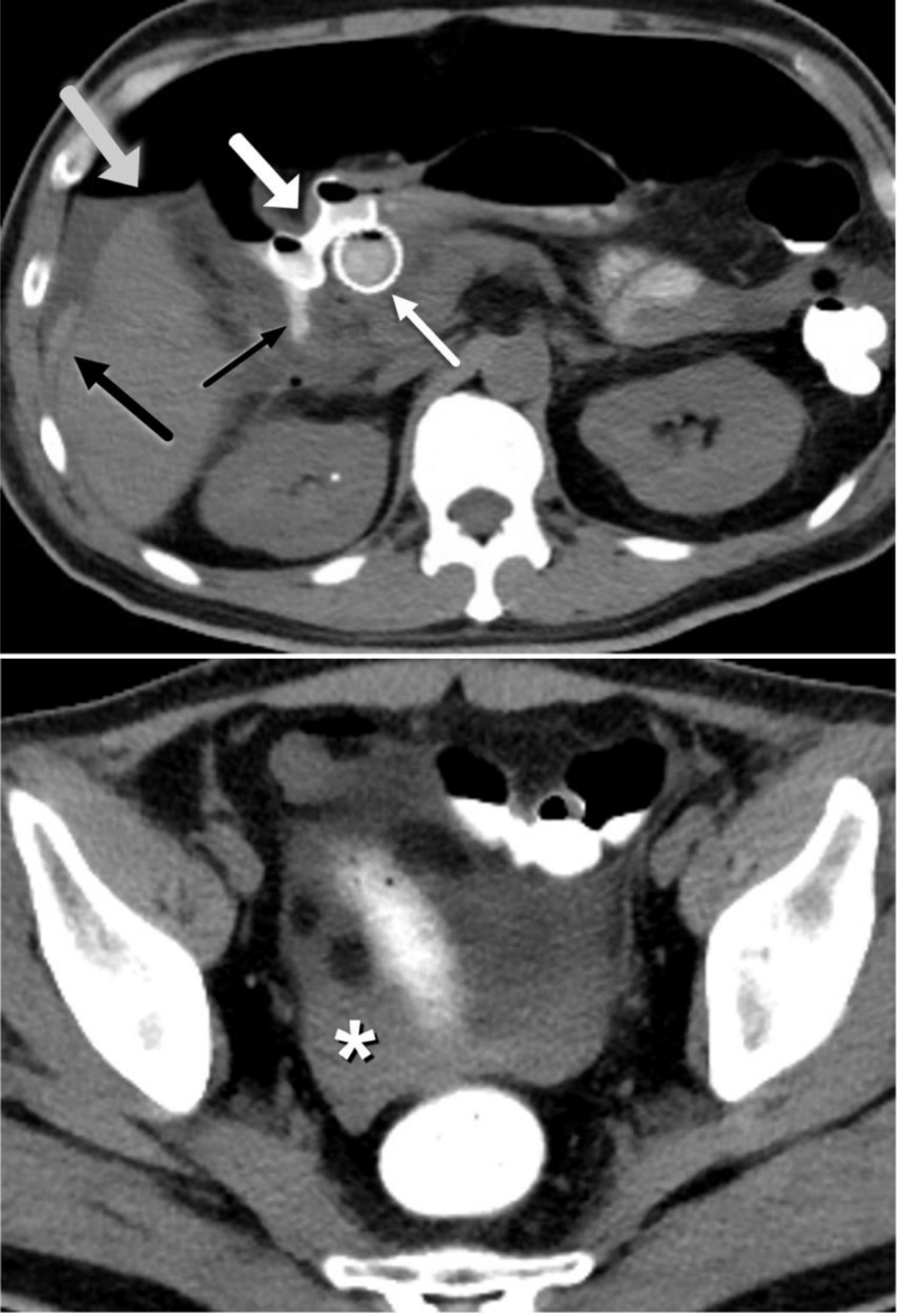
73 year-old male with pancreatic carcinoma and acute cholecystitis. **A**. Axial CT image demonstrates LAMS-cholecystogastrostomy (thick white arrow) performed in attempt to relieve acute cholecystitis. Axios maldeployment or dislodgement from gallbladder is evident with oral contrast (thick black arrow) noted in the perihepatic space, as well as air-fluid level (gray arrow) indicating presence of pneumoperitoneum and ascites. Extraluminal contrast leakage from the distal portion of the LAMS is noted (thin black arrow). Biliary stent containing contrast and small amount of air (thin white arrow) is noted in the CBD. **B**. Axial CT image shows fluid-fluid level in pelvic ascites indicating presence of extravasated oral contrast mixing with fluid (white asterisk)

**Fig. 5:**
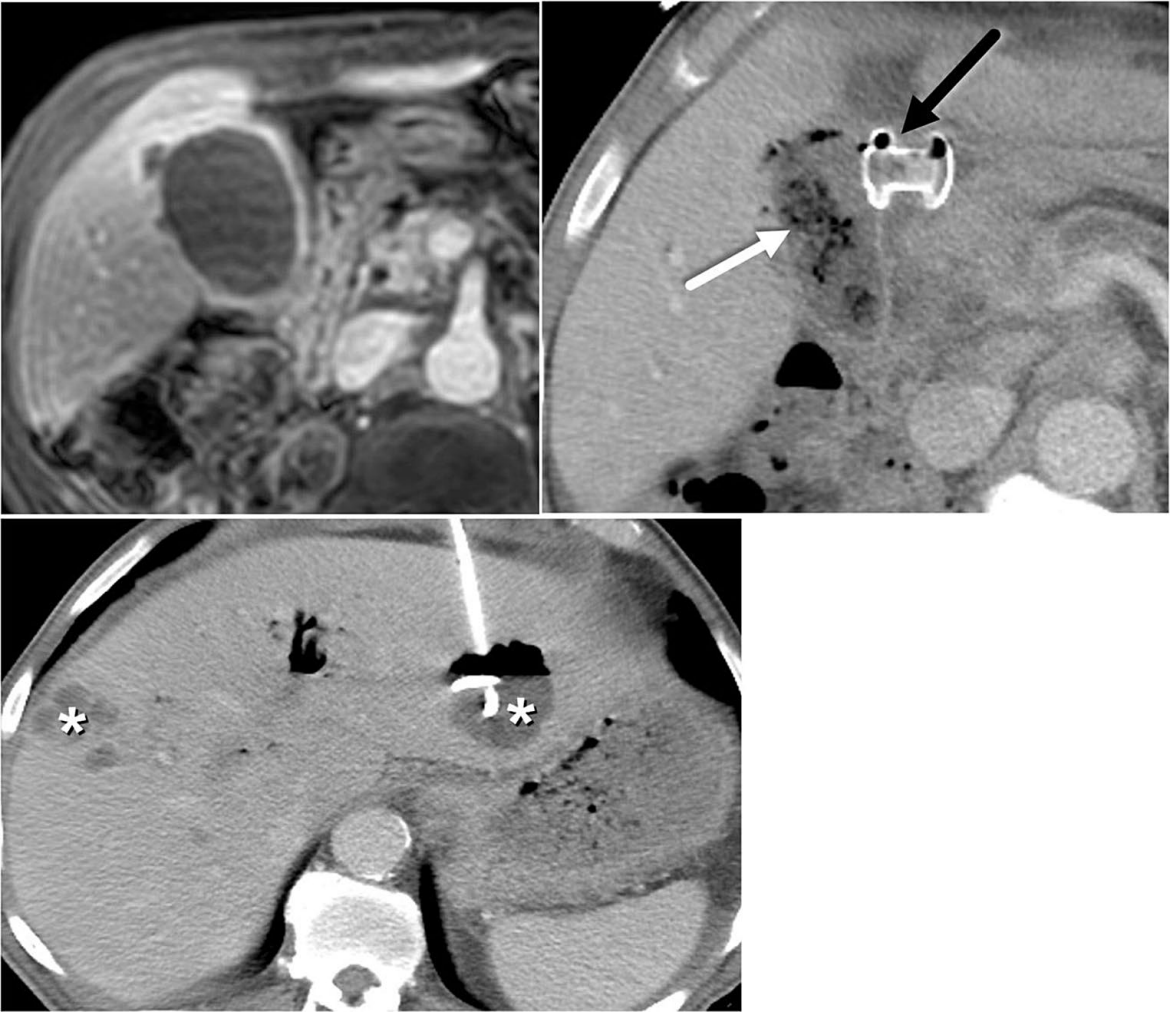
72 year-old female with pancreatic neck cancer and acute cholecystitis. **A**. Post-contrast axial LAVA MRI shows irregular gallbladder wall with focal outpouchings indicating contained gallbladder wall perforation (white arrows) related to gangrenous cholecystitis, a contraindication to endoscopic gallbladder drainage, which was not recognized prospectively. **B**. LAMS cholecystogastrostomy was subsequently placed (black arrow). Expected air in the partly decompressed gallbladder is noted (white arrow). **C**. The patient developed multiple hepatic abscesses (white asterisks) requiring percutaneous drainage, and ultimately succumbed to sepsis

With the growing use of LAMS for endoscopic gallbladder drainage supported by its recent FDA approval, radiologists should be aware of its respective associated risks, contraindications, and complications. A meta-analysis including 393 patients using LAMS for EUS-GBD demonstrated an overall adverse event rate of 12.7%, including bleeding, stent migration, stent occlusion, recurrent cholecystitis or cholangitis, bile leak, perforation (duodenal or gallbladder), and death (Fig. 4) [22]. One of the contraindications to EUS-GBD is gangrenous or perforated cholecystitis due to increased risk of leak, and abscess formation owing to gallbladder wall friability (Fig. 5). An important consideration for candidates for endoscopic gallbladder drainage, who may undergo liver transplantation in the future, is that ET-GBD is preferred in this cohort as it allows for preservation of native biliary anatomy and structural integrity of the gallbladder [15]. Because of these reasons, it is recommended that patients considered for endoscopic gallbladder drainage undergo multidisciplinary evaluation.

## Choledochoduodenostomy or choledochogastrostomy for biliary drainage

ERCP with transpapillary stenting using self-expandable metallic stents (SEMS) remains the first line treatment for biliary drainage of malignant biliary obstruction (MBO) and is commonly utilized to resolve jaundice in patients with benign common bile duct strictures. However, in cases of a high grade CBD stricture due to ductal or papillary encasement/invasion by tumor, duodenal obstruction, periampullary diverticulum, or prior duodenal stenting across the papilla, biliary access via ERCP may not be possible [23, 24]. When ERCP fails, percutaneous transhepatic biliary drainage (PTBD) is standard rescue therapy with high clinical success rates, but also associated with a high rate of adverse events and reduced quality of life [25].

**Fig. 6:**
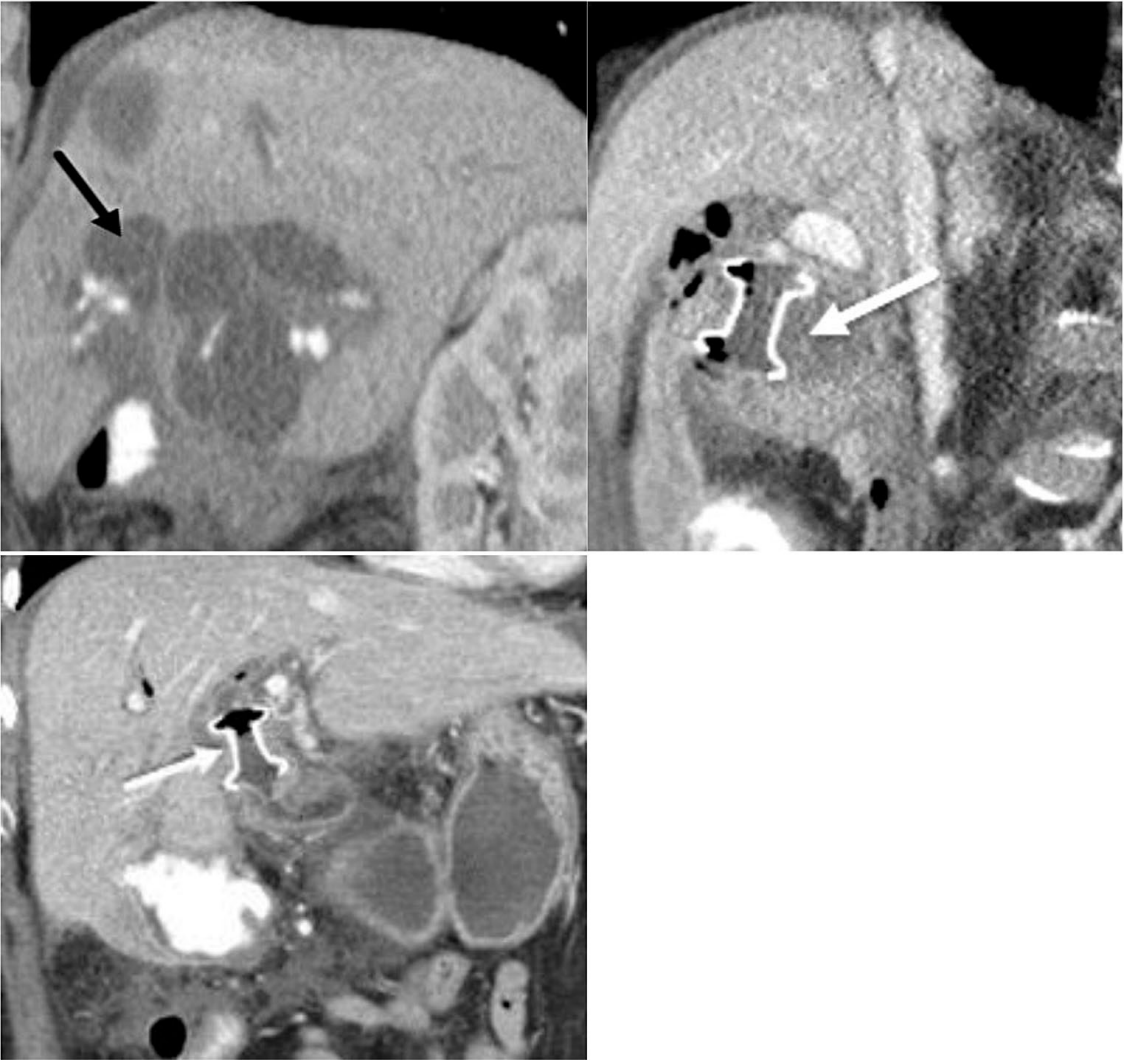
63 year-old woman with a large pancreatic head adenocarcinoma obstructing CBD, which could not be stented via transpapillary approach. **A**. Contrast-enhanced sagittal CT image demonstrates severe intrahepatic biliary ductal dilatation (black arrow). **B** and **C**. Contrast-enhanced sagittal **(B)** and coronal **(C)** CT images show LAMS (white arrow) placed between the duodenal bulb and proximal CBD with resolved intrahepatic biliary ductal dilatation and expected pneumobilia

Endoscopic ultrasound-guided biliary drainage (EUS-BD), such as EUS-guided choledochoduodenostomy (EUS-CD) involving extra-anatomic stent placement from the duodenum into the bile duct, has emerged as a viable option to PTBD, demonstrating high clinical success rates and fewer recurrences [26–31] (Fig. 6).

A meta-analysis of 284 patients by Krishnamoorthi et al. demonstrated 95.7% technical success and a 95.9% clinical success with a pooled adverse event rate of 5.2%, which is statistically similar to that of SEMS [32]. AEs involved perforation, bile leak, bleeding, cholangitis, and abdominal pain. However, there is no current consensus on adverse event rates, as other studies cite a significantly higher number, with another study citing a 36.8% complication rate [28]. Comparative studies with further analysis of adverse events and long-term clinical success are pending. Two recent RCTs have demonstrated EUS-guided choledochoduodenostomy as an alternative to ERCP as first-line treatment of malignant distal biliary obstruction, particularly in unresectable patients [33, 34].

## Conduit creation in patients with surgically altered anatomy

**Fig. 7:**
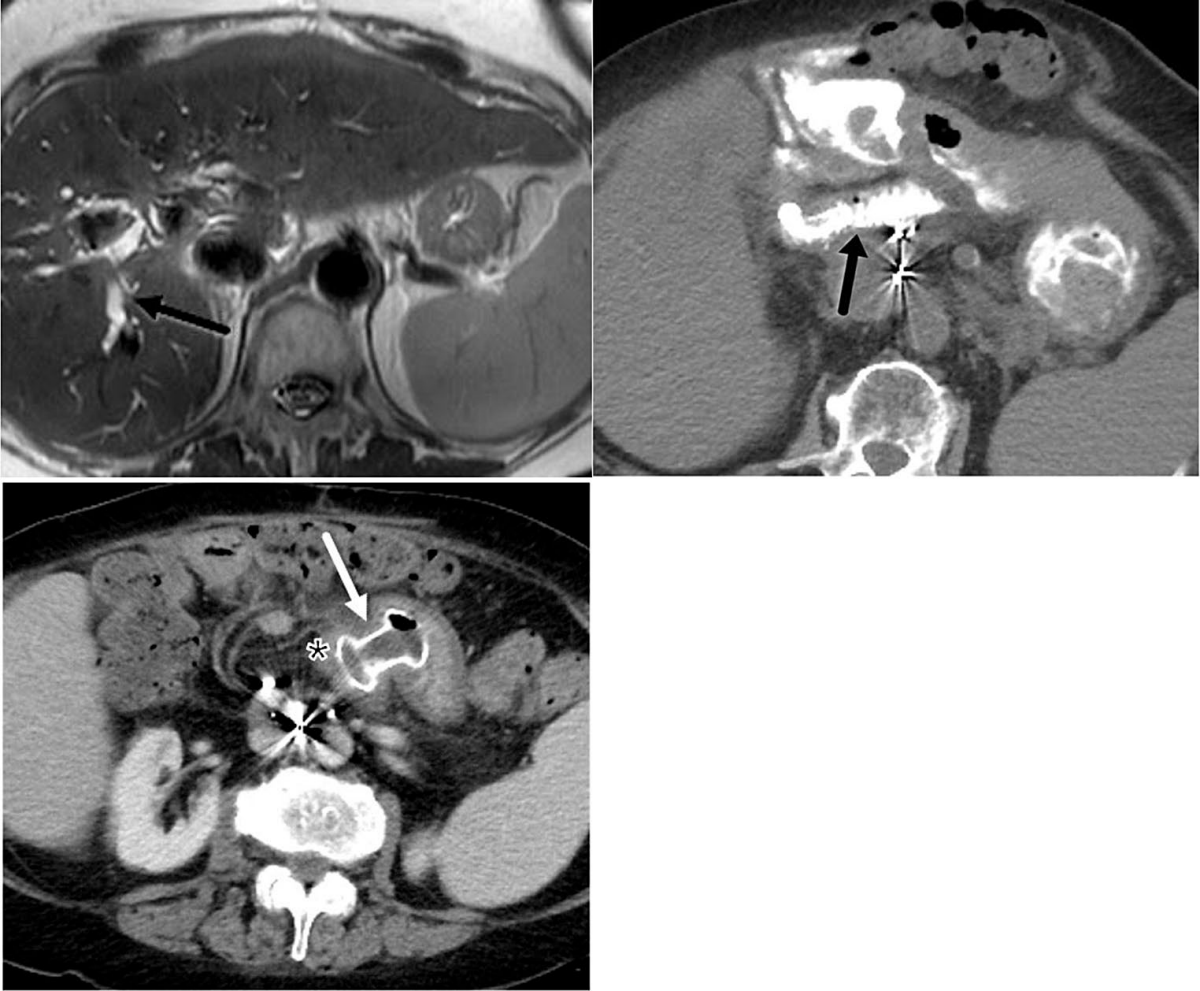
76 year-old female with history of Whipple surgery for malignant IPMN developed biliary strictures after chemotherapy and radiation therapy. The patient required technically challenging ERCPs for biliary stricture dilatation every several months due to the recurrent nature of biliary strictures and recurrent cholangitis. **A**. Axial T2-weighted MRI shows intrahepatic biliary dilatation (black arrow) requiring repeated stricture dilatations and frequent stent exchanges every 2–3 three months. Frequent ERCPs were technically challenging due to surgically altered anatomy after Whipple surgery. A proposed intervention included creating a permanent conduit traversing the gastrojejunostomy between the remnant stomach and the pancreaticobiliary limb to allow ease of endoscopic access for biliary dilatation/drainage. **B**. To promote placement of LAMS across the gastrojejunostomy, the pancreaticobiliary limb had to be distended prior to LAMS deployment by contrast introduced via percutaneous transhepatic cholangiogram through hepaticojejunostomy. This axial CT image demonstrates distention of the pancreaticobiliary limb with contrast (black arrow). **C**. Contrast-enhanced axial CT confirms that the LAMS (white arrow) was successfully placed between the stomach and pancreaticobiliary limb (asterisk) to create a permanent indwelling conduit for ERCP access

Various abdominal surgeries can disrupt normal contiguity of the GI tract, potentially precluding or complicating future endoscopic interventions, such as ERCP access. These postoperative anatomic variations are most frequently encountered and reported in the literature in bariatric patients following Roux-en-Y gastric bypass (RYGB). They are also seen in patients with Billroth II gastrectomy, pancreaticoduodenectomy (Whipple procedure), and Roux-en-Y hepaticojejunostomy. In these scenarios, LAMS can serve as a conduit between the stomach and an excluded portion of the GI tract and can facilitate therapeutic procedures in patients with altered surgical anatomy. We demonstrate a case of LAMS placement to facilitate endoscopic access for repeat ERCP every three months in a patient with a history of Whipple surgery and refractory biliary strictures causing recurrent cholangitis (Fig. 7).

Roux-en-Y gastric bypass surgery significantly alters the anatomy of the upper GI tract, restricting endoscopic access to the distal stomach, duodenum, biliary tree, and pancreas. Rapid post-surgical weight loss increases the risk of cholelithiasis and choledocholithiasis, which may necessitate access to the duodenum to perform ERCP for biliary decompression [35].

Three options currently exist for performing ERCP in post-RYGB patients. Two traditional approaches include double-balloon enteroscopy and laparoscopy-assisted ERCP, but both techniques have significant limitations. For example, double-balloon enteroscopy-assisted ERCP has only about a 50% success rate even in the hands of experienced endoscopists, and LA-ERCP carries operative risks [36, 37]. The third, more recent approach is called gastric access temporary for endoscopy (GATE), which involves creating temporary transgastric access via a LAMS for subsequent endoscopy, including ERCP, EUS, mucosal resection, endoscopic submucosal dissection and biopsies [38]. Subsequent ERCP is performed through the conduit, and is commonly referred to as EDGE (Endoscopic Ultrasound-Directed transgastric ERCP). In brief, the procedure is performed in two stages: using endosonographic guidance the excluded stomach is located from the gastric pouch and LAMS is deployed between the two structures [39]. Except for in highly urgent situations like acute cholangitis, subsequent intervention through LAMS is typically delayed for two weeks to promote fistula maturation and to mitigate the risk of LAMS dislodgement from endoscope passage. Once the desired endoscopic procedure is complete, the LAMS can be removed, and the resultant fistula may be closed endoscopically.

Chiang et al. described results of 66 patients undergoing EDGE, achieving a 92% technical success rate. However there was a 20% adverse event rate, including bleeding, malpositioning, migration, perforation, and pancreatitis. A majority of complications occurred after using transgastric access, so the study concluded that GATE via transjejunal access may be safer [40]. A comparative meta-analysis of 1,268 RYGB patients who underwent either the EDGE procedure vs. LA-ERCP reported comparable technical (95.5% vs. 95.9%) and clinical (95.5% and 92.9%) success rates. AE rates were high, but comparable (21.9% vs. 17.4%), including stent migration due to immature fistula formation and manipulation via duodenoscope, as well as bleeding, perforation, and infection [41].

## Endoscopic gastrojejunostomy bypass

**Fig. 8:**
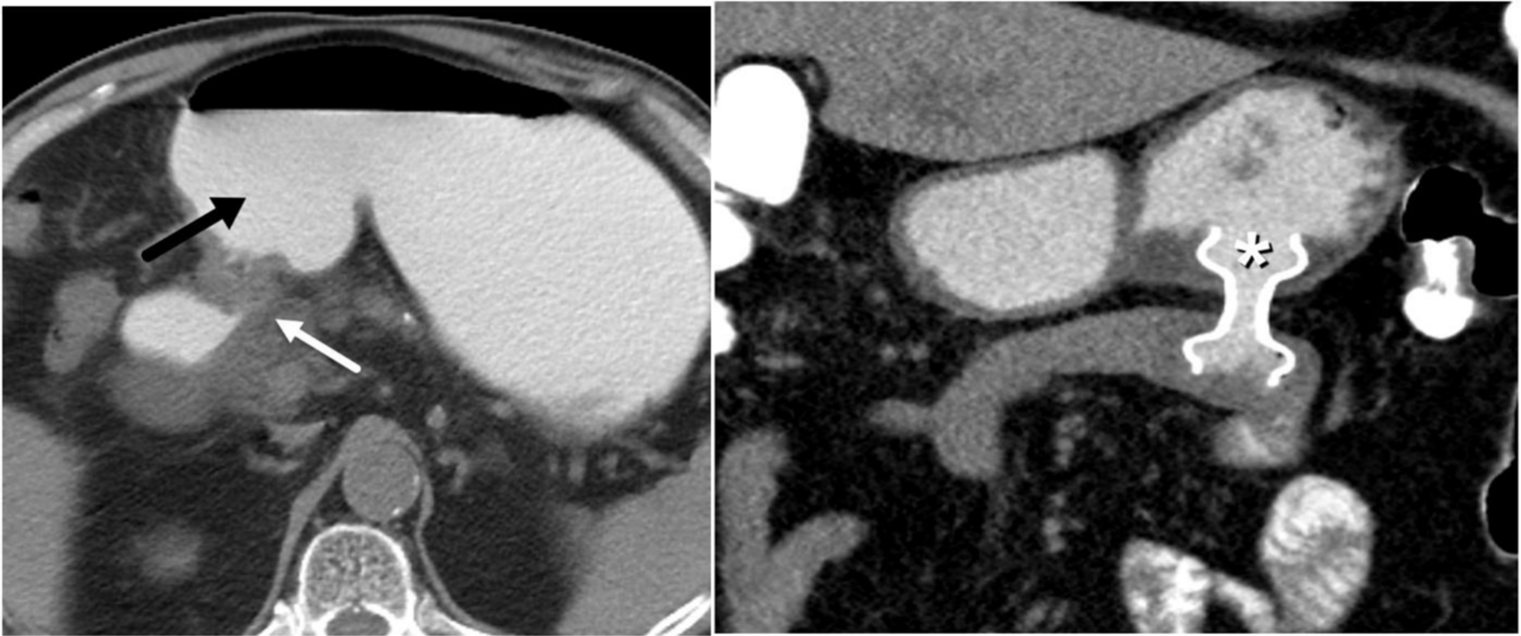
83 year-old male with pancreatic carcinoma receiving palliative endoscopic gastrojejunostomy. **A**. Contrast-enhanced axial CT image showing partial gastric outlet obstruction with distended stomach filled with oral contrast (black arrow) due to known pancreatic carcinoma (white arrow) at pylorus and first portion of duodenum. **B**. Contrast-enhanced coronal CT image shows palliative gastrojejunostomy via LAMS (white asterisk) demonstrating luminal patency that relieved obstruction and prevented need for surgery

Gastric outlet obstruction (GOO), a syndrome associated with substantial morbidity, including abdominal pain, early satiety, postprandial vomiting, and weight loss, can result from both benign (peptic ulcer disease, polyps, strictures, etc.) and malignant (tumors involving gastric antrum, pylorus, pancreatic head and proximal duodenum) etiologies [42]. Surgical gastrojejunostomy (SGJ) and endoscopic interventions, including balloon dilation and luminal stenting are primary treatments for this syndrome, but they are limited by surgical morbidity up to 40% and recurrent obstruction in 20–30% after endoscopic stenting [43, 44]. EUS-guided gastrojejunostomy (EUS-GJ, also known as EUS-GE) with LAMS has recently emerged as a non-operative alternative in the treatment of GOO, especially in high-risk surgical patients with malignant obstruction. EUS-GJ is performed by endosonographically locating a portion of the jejunum distal to the mechanical obstruction from the stomach lumen. LAMS is then deployed to create a gastrojejunal anastomosis, thereby bypassing the obstruction (Fig. 8). A similar tract can be created using LAMS through a retrograde approach, where the EUS scope can be advanced beyond the mild luminal obstruction, and LAMS is deployed from the small bowel to the stomach [45].

A meta-analysis of twelve studies of EUS-GJ procedures including 285 patients utilizing LAMS in both benign and malignant GOO revealed a technical and clinical success rate of 92% and 90%, respectively, and a recurrence of symptoms in 9% of patients and an adverse event rate of 12% [46]. In the first randomized controlled trial between laparoscopic gastrojejunostomy (Lap-GJ) and EUS-GJ, Perez-Miranda et al. found no statistically significant differences in technical and clinical success rates. However, AE rates differed significantly, with AEs occurring in 41% of Lap-GJ patients and 12% of EUS-GJ patients. It was noted that EUS-GJ could be offered to most patients and thus could be a widely considered minimally invasive treatment alternative [47]. An international, multicenter retrospective study of 77 patients found comparable technical and clinical success, but significantly reduced time to oral intake, shorter median hospital stays, and lowered adverse event rates in EUS-GJ, identifying it as the preferred modality [48].

## Benign gastrointestinal strictures

Current interventional treatments for gastrointestinal strictures include serial balloon dilations with or without steroid injections, stenting, and endoscopic electrocautery incision therapy [49–52]. Depending on the etiology of the stricture, some strictures may require multiple treatments [53–56]. Temporary placement of self-expanding metal stents (SEMS), an off-label use, has been successfully used to reduce risk of stricture recurrence. However, SEMS are associated with migration rates up to 30–40% [57–60]. The bi-flanged design of LAMS improves anchorage, mitigating the risk of migration seen with SEMS. LAMS have been utilized in patients with short, benign strictures throughout the gastrointestinal tract, including perianastomotic strictures, and strictures related to radiation therapy, caustic injury, peptic disease, and chronic pancreatitis [61].

LAMS have specifically shown high technical and clinical success rates for the treatment of gastrojejunal anastomotic strictures after RYGB. A meta-analysis by Tan et al. comprising 6 studies totaling 144 patients reported a technical success rate of 97%, with clinical success rate of 74%. Clinical success varied by stricture location between esophagogastric (64%), gastroduodenal (67%), gastrojejunal (78%), pylorus (78%), and colonic strictures (85%). A 30% adverse event rate was observed with an 11% migration rate and a 7% new stricture rate [61]. To date, there are no comparative studies between LAMS with balloon dilation or use of other stents for benign strictures.

**Fig. 9:**
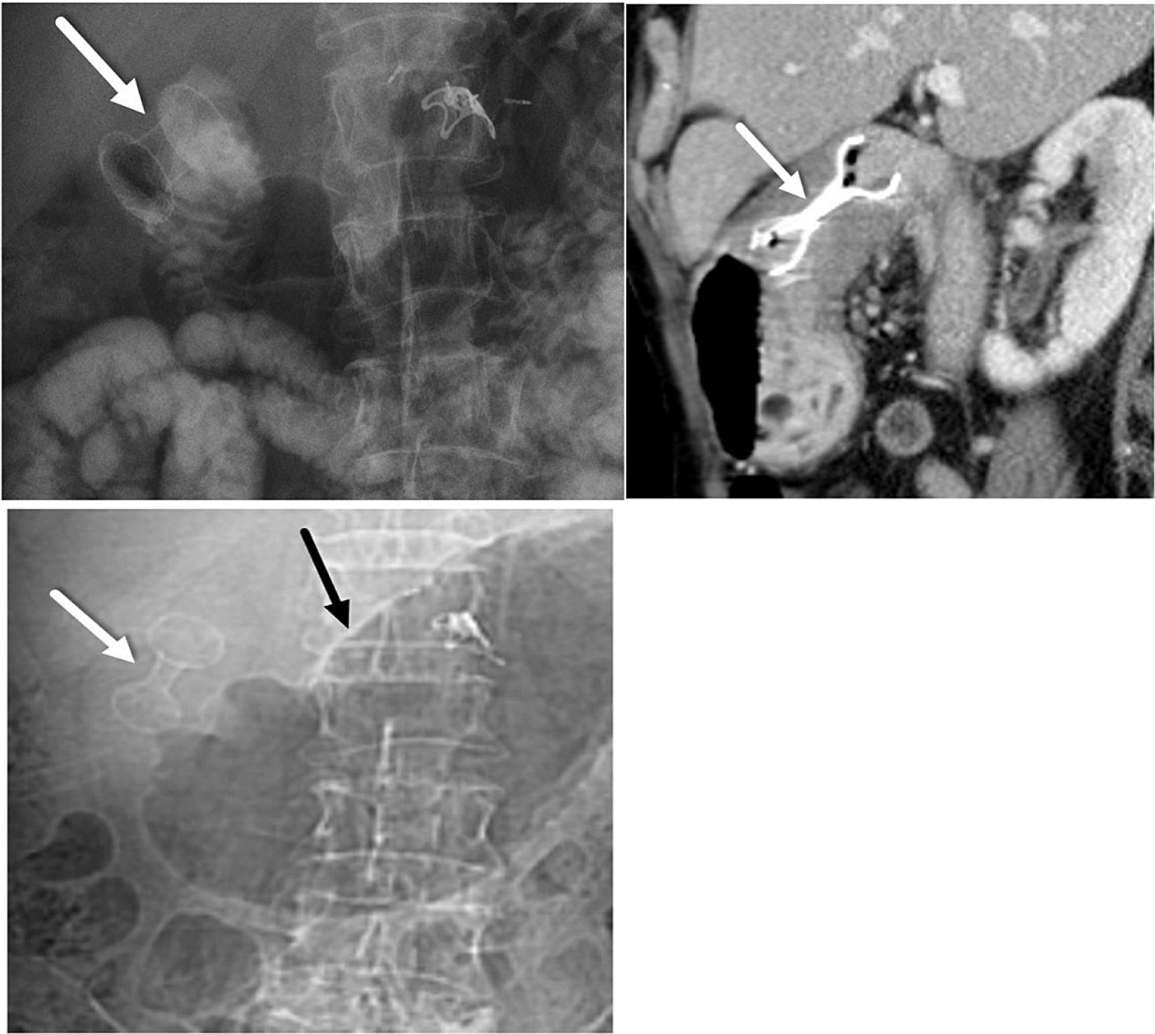
63 year-old patient presenting with benign pyloric strictures secondary to peptic ulcer disease leading to gastric outlet syndrome. **A.** Abdominal radiograph shows intraluminal LAMS (white arrow) that was placed to relieve a benign pyloric stricture with significant clinical improvement. **B.** Contrast-enhanced sagittal CT image obtained a year later, when the patient returned after being lost to follow-up, demonstrates new luminal narrowing (white arrow) due to mucosal ingrowth of LAMS placed across the pylorus. **C.** A frontal CT topogram obtained at the same time when the patient represented demonstrates new narrowing (white arrow) of the LAMS lumen as a complication due to mucosal ingrowth and resultant recurrent gastric outlet obstruction (dilated stomach (black arrow))

Stents used to treat malignant conditions are typically left indefinitely, while stents placed for benign strictures are usually removed within a few weeks. SEMS for benign strictures are typically removed within two weeks, but this can lead to high stricture recurrence rates and need for reintervention [62, 63]. The optimal indwelling time for LAMS has not been established; however, higher median indwelling times of LAMS ranging from 60 to 107 days have been reported, indicating promise in reducing recurrence rates and repeat interventions [64–67]. We report an instance of a patient with initial improvement in gastric outlet obstruction after a benign pyloric stricture caused by peptic ulcer disease was treated with LAMS. The patient, who was subsequently lost to follow-up for a year, returned with a recurrent gastric outlet obstruction, now due to mucosal ingrowth of LAMS causing obstruction (Fig. 9).

## Drainage of postoperative and pelvic collections

Postsurgical fluid collections (PSFCs) have traditionally been drained percutaneously when accessible or require surgical drainage (89). Recently, EUS-guided drainage has emerged as an effective minimally invasive alternative to these standard approaches.

**Fig. 10:**
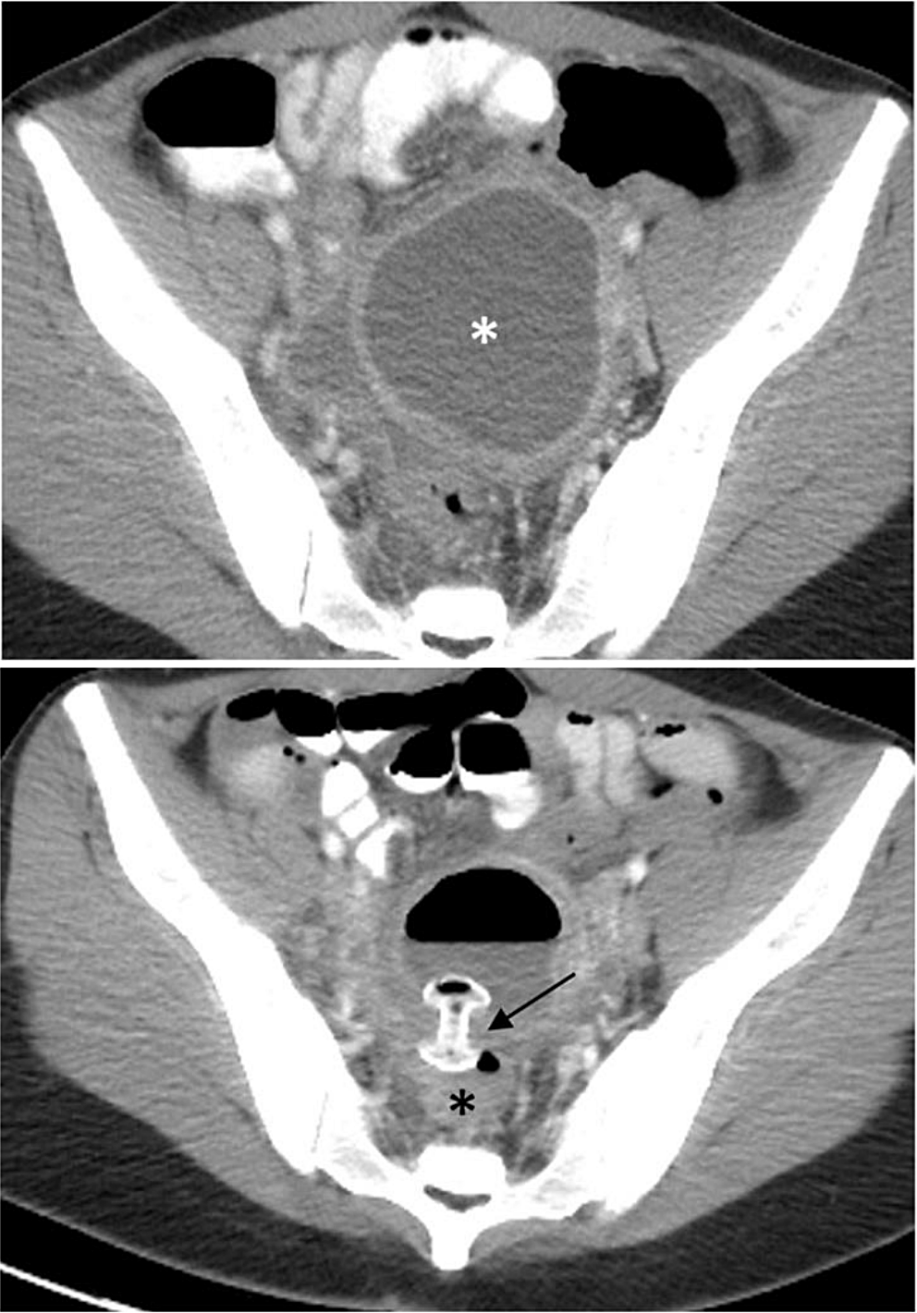
26 year-old female presented with ruptured tubo-ovarian abscess that was inaccessible by IR drainage. **A**. Contrast-enhanced axial CT image demonstrates ruptured tubo-ovarian abscess (white asterisk). **B**. Contrast-enhanced axial CT image demonstrates successful interval drainage into rectum (black asterisk) via LAMS (black arrow)

More recently, EUS-guided transluminal drainage via LAMS has been used to successfully drain well-defined fluid collections following distal pancreatectomies, splenectomies, hepatectomies [68, 69]. In the largest multicenter study to date on the use of LAMS for postoperative surgical collections, involving 62 patients, Yang et al. reported technical and clinical success rates were 96.8% and 91.9%, respectively, with intraprocedural and postprocedural adverse event rates were 1.6% and 11.3%, respectively [70]. Collections caused by pelvic inflammatory disease, diverticulitis, appendicitis, and anastomotic leaks have also been successfully drained via LAMS (Fig. 10). Similar to pancreatic fluid collections (PFCs), these collections should have a well-defined wall, be at least 4 cm in diameter, lie within 1 cm of the GI lumen, and lack intervening vessels. Contraindications to endoscopic drainage via LAMS include ascites, active inflammatory disease of the GI tract, and neoplastic involvement of the bowel that would be traversed by the stent (2). No prospective studies comparing EUS-drainage using LAMS to other drainage techniques of postoperative and pelvic fluid collections have been published thus far. However, current studies show promising alternative to percutaneous or surgical drainage.

## Imaging of LAMS-related adverse events

Complications of LAMS vary based on their anatomic location and intended use. The bi-flanged design and close apposition of mucosal walls offered by LAMS have encouraged endoscopists to utilize it more frequently for creating more controlled, full-thickness mucosal tracts, even beyond the novel applications discussed in this paper. A review that pooled data on LAMS use for peripancreatic fluid collection, bile duct, and gallbladder drainage identified bleeding, stent migration or dislodgment, perforation, and occlusion as the most common adverse events reported [71]. In general, complications of LAMS are related to the inherent instability of stents, manipulation and instrumentation of the walls of the gastrointestinal tract and disruption of wall integrity.

**Fig. 11:**
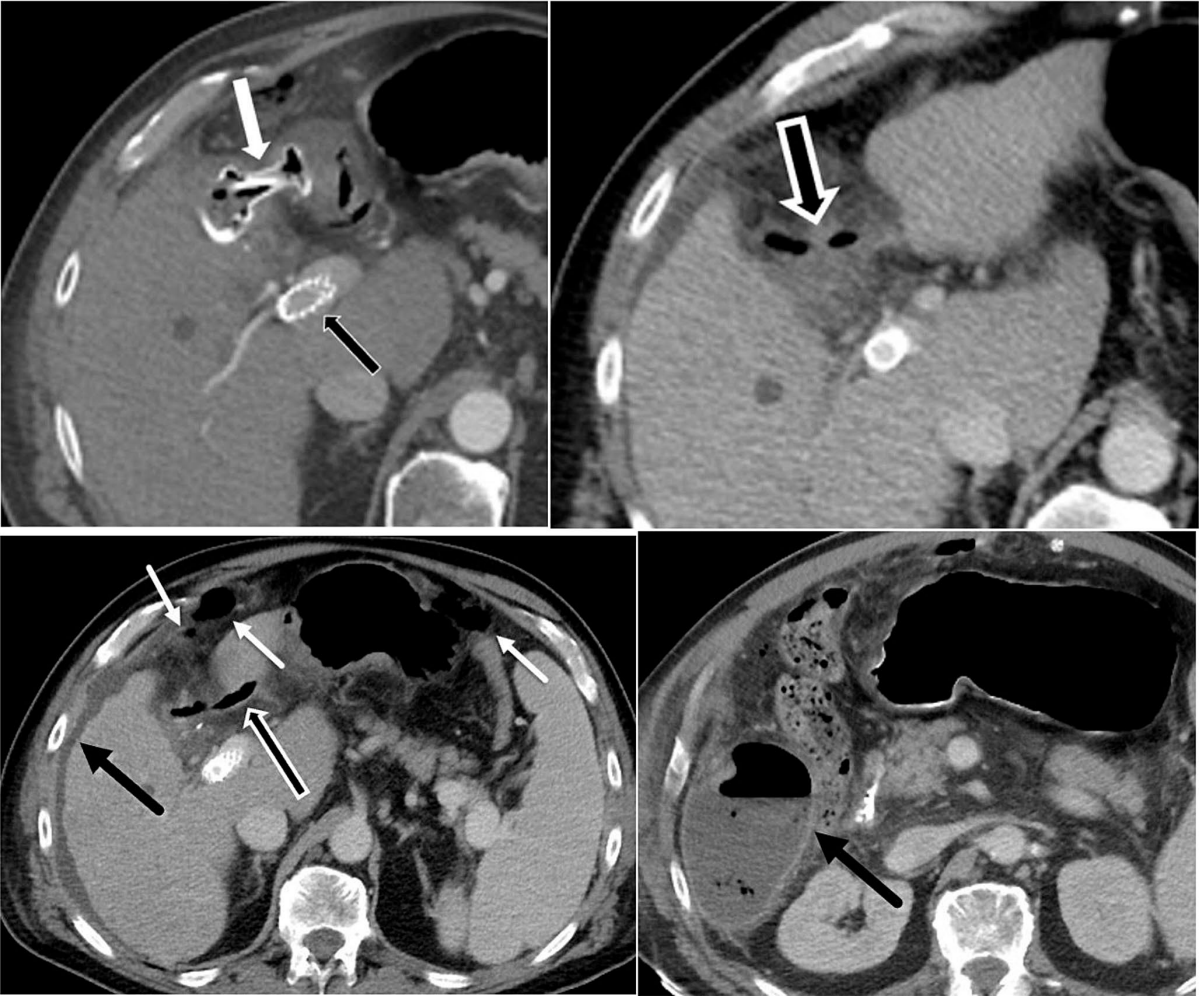
58 year-old cirrhotic male patient with acute on chronic cholecystitis complicated by portal hypertension s/p TIPS procedure. **A**. Contrast-enhanced axial CT image demonstrates LAMS (white arrow) placement between stomach and gallbladder with a pigtail catheter across LAMS facilitating anchorage, and TIPS (black arrow). **B**. One month later, interval laparoscopic cholecystectomy has taken place with removal of Axios stent. Contrast-enhanced axial CT demonstrates visualization of a residual fistulous tract (black arrow). **C**. A few days later, contrast-enhanced axial CT demonstrates persistent fistulous tract (outlined black arrow) leading to accumulation of free air (white arrows) and fluid in the upper and mid abdomen (black arrow). **D**. Three weeks later, contrast-enhanced axial CT image demonstrates interval development of right subhepatic abscess (black arrow)

**Fig. 12:**
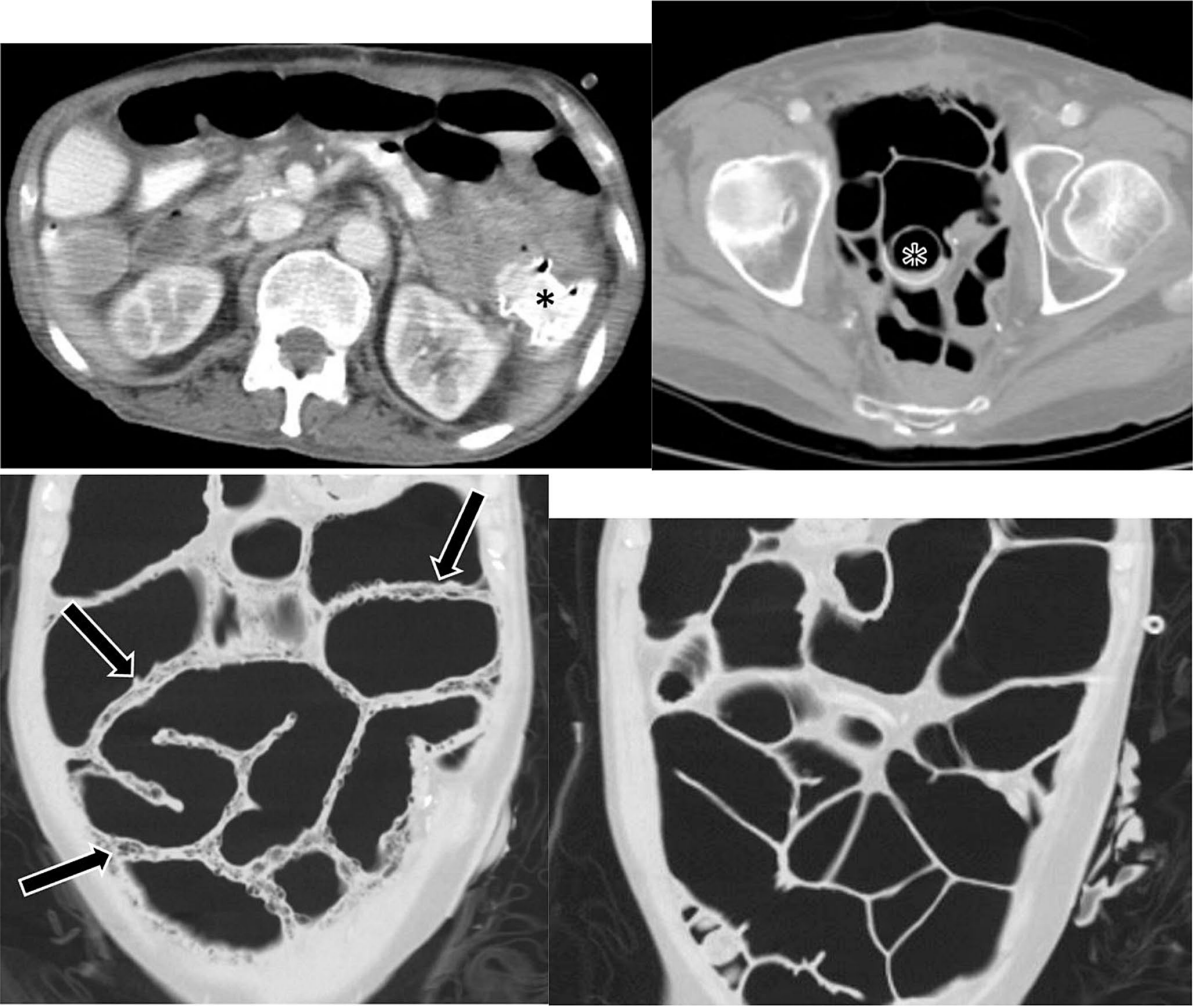
58 year-old man with history of distal rectal cancer, prior abdominoperineal resection and subsequent pelvic exenteration due to local recurrence. Patient’s course was complicated by chronic small bowel obstruction due to empty pelvis syndrome and recurrent pelvic disease. LAMS was placed between small bowel and descending colon through colostomy access to allow bypass and decompression. **A**. Contrast-enhanced axial CT demonstrates LAMS (black asterisk) in place in left upper quadrant bridging the jejunum and descending colon. **B**. Contrast-enhanced axial CT three months later demonstrates dislodged LAMS (black asterisk), now visualized in distal small bowel in the pelvis. **C**. Coronal CT image demonstrates new, extensive pneumatosis intestinalis (black arrows) and small bowel dilatation. The patient was relatively asymptomatic with no evidence of infection or ischemia on laboratory studies. One possible hypothesis is that dislodgement of LAMS may have resulted in an enterocolic fistula with resultant intramural escape/dissection of bowel gas into the bowel wall. **D**. Coronal CT image obtained three weeks after replacement of LAMS demonstrates complete resolution of pneumatosis intestinalis

One of the specific complications that can occasionally be encountered following LAMS removal or dislodgement is inadvertent fistulous tract formation. Devices commonly used to close transmural defects after LAMS removal are through-the-scope-clips (TTSC), over-the scope clips (OTSC), and endoscopic suturing. Although these techniques are usually successful, occasionally entero-biliary or entero-enteric fistula formation may occur and be associated with a persistent leak (Fig. 11). Additionally, we report an unusual case of LAMS dislodgement between the small bowel and descending colon, initially placed to bypass small bowel obstruction due to empty pelvis syndrome and local recurrence. This resulted in enterocolic fistula development with subsequent diffuse pneumatosis intestinalis, which resolved after LAMS replacement (Fig. 12).

With increasing utilization of LAMS, it is crucial that radiologists recognize common complications associated with LAMS, particularly pseudoaneurysms, fistulas, stent migration, leaks, potential tissue ingrowth, and perforation. As detailed earlier, although several studies suggest that LAMS can remain indwelling longer than other stents, there is still no data regarding ideal indwelling time. Therefore, radiologists must remain vigilant to potential delayed adverse events.

## Summary

Since their introduction, LAMS have revolutionized the field of gastroenterology and advanced endoscopy, markedly expanding the variety of therapeutic endoscopic procedures. The primary FDA-approved usage of LAMS for drainage of peripancreatic fluid collections was recently supplemented by an additional indication for endoscopic gallbladder drainage. Moreover, they are increasingly being utilized as minimally invasive alternatives to surgery for the treating malignant biliary disease, creating palliative enterostomies, facilitating interventional procedures in patients with surgically altered anatomy, and managing various abdominopelvic obstructions and collections. As LAMS applications and clinical experience continue to expand, familiarity with the various on- and off-label applications and appearance of LAMS will allow radiologists to better recognize the different clinical scenarios of its use, associated adverse events, and collaborate in multidisciplinary teams to support patient care.

## Electronic supplementary material

Below is the link to the electronic supplementary material.

Supplementary Material 1

## Data Availability

No datasets were generated or analysed during the current study.
