# Supplementary material for: A stent of strength: use of lumen-apposing metal stents (LAMS) for biliary pathologies and other novel applications
Source: Abdom Radiol (NY). 2024 Sep 10;50(2):902–15. doi: 10.1007/s00261-024-04561-9 (PMC11794341; doi:10.1007/s00261-024-04561-9)
Supplement: Supplementary file 1 — Supplementary Material 1 [file 261_2024_4561_MOESM1_ESM.pptx]

## Slide 1
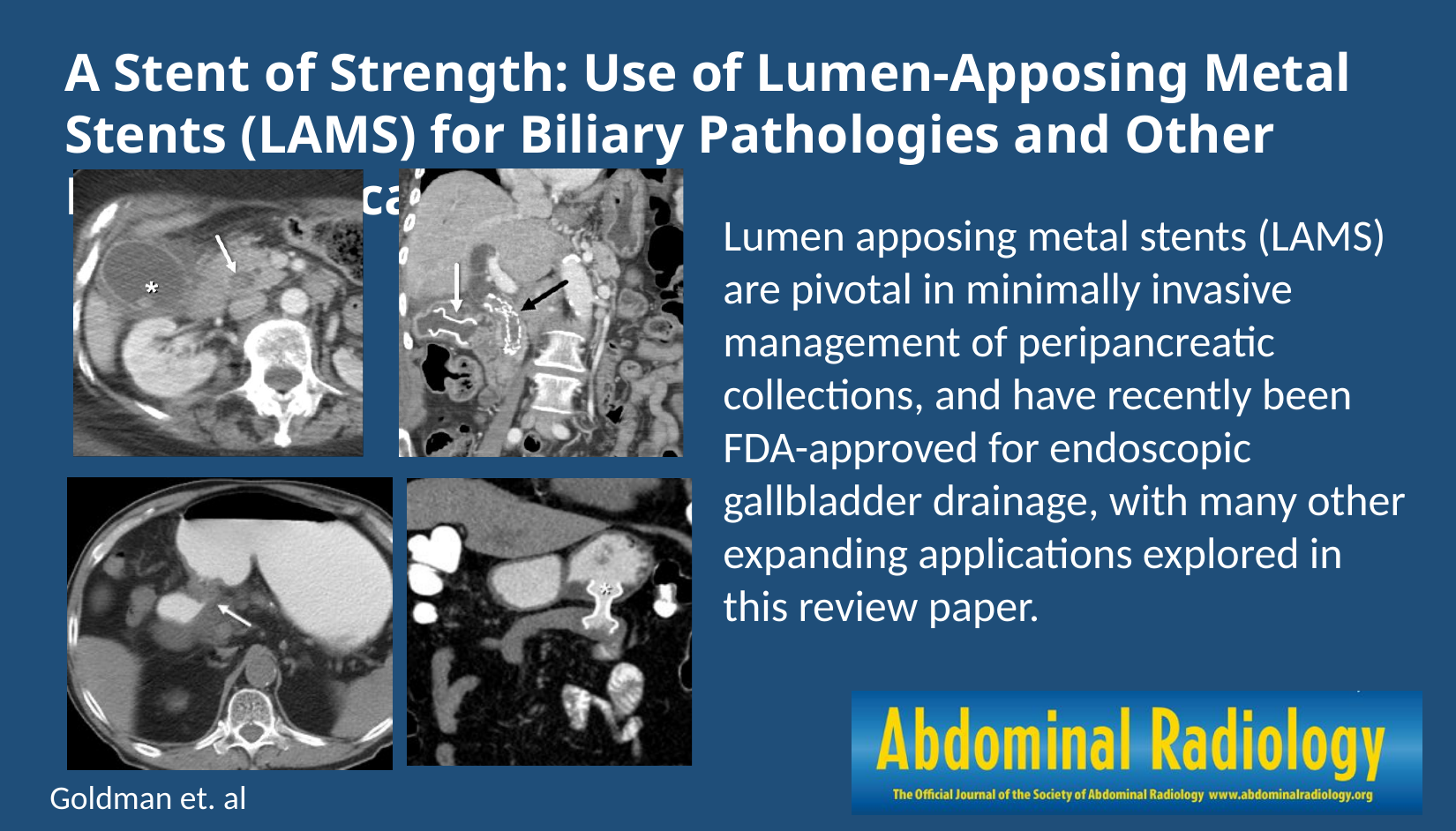

A Stent of Strength: Use of Lumen-Apposing Metal Stents (LAMS) for Biliary Pathologies and Other Novel Applications
Lumen apposing metal stents (LAMS) are pivotal in minimally invasive management of peripancreatic collections, and have recently been FDA-approved for endoscopic gallbladder drainage, with many other expanding applications explored in this review paper.
Goldman et. al
